# Health Outcomes in US Children with Abdominal Pain at Major Emergency Departments Associated with Race and Socioeconomic Status

**DOI:** 10.1371/journal.pone.0132758

**Published:** 2015-08-12

**Authors:** Louise Wang, Corinna Haberland, Cary Thurm, Jay Bhattacharya, K. T. Park

**Affiliations:** 1 School of Medicine, Stanford University, Stanford, CA, United States of America; 2 Department of Health Research and Policy, Stanford University School of Medicine, Stanford, CA, United States of America; 3 Children’s Hospital Association, Overland Park, KS, United States of America; 4 Center for Health Policy/ Primary Care Outcomes Research, Stanford University, Stanford, CA, United States of America; 5 Department of Medicine, Stanford University School of Medicine, Stanford, CA, United States of America; 6 Department of Economics, Stanford University, Stanford, CA, United States of America; 7 Division of Gastroenterology, Hepatology, and Nutrition, Department of Pediatrics, Stanford University School of Medicine, Stanford, CA, United States of America; Yokohama City University, JAPAN

## Abstract

**Objective:**

Over 9.6 million ED visits occur annually for abdominal pain in the US, but little is known about the medical outcomes of these patients based on demographics. We aimed to identify disparities in outcomes among children presenting to the ED with abdominal pain linked to race and SES.

**Methods:**

Data from 4.2 million pediatric encounters of abdominal pain were analyzed from 43 tertiary US children’s hospitals, including 2.0 million encounters in the emergency department during 2004-2011. Abdominal pain was categorized as functional or organic abdominal pain. Appendicitis (with and without perforation) was used as a surrogate for abdominal pain requiring emergent care. Multivariate analysis estimated likelihood of hospitalizations, radiologic imaging, ICU admissions, appendicitis, appendicitis with perforation, and time to surgery and hospital discharge.

**Results:**

Black and low income children had increased odds of perforated appendicitis (aOR, 1.42, 95% CI, 1.32- 1.53; aOR, 1.20, 95% CI 1.14 – 1.25). Blacks had increased odds of an ICU admission (aOR, 1.92, 95% CI 1.53 - 2.42) and longer lengths of stay (aHR, 0.91, 95% CI 0.86 – 0.96) than Whites. Minorities and low income also had lower rates of imaging for their appendicitis, including CT scans. The combined effect of race and income on perforated appendicitis, hospitalization, and time to surgery was greater than either separately.

**Conclusions:**

Based on race and SES, disparity of health outcomes exists in the acute ED setting among children presenting with abdominal pain, with differences in appendicitis with perforation, length of stay, and time until surgery.

## Introduction

Abdominal pain is the most common gastrointestinal (GI) symptom in the United States (US), with an estimated 15.9 million annual all-case outpatient encounters [1], including over 900,000 visits each year to the emergency department (ED) by US children less than 15 years of age [2]. In the 2009 data from the National Hospital Ambulatory Medical Care Survey, among all ED abdominal pain cases, 50% were found to require urgent or emergent care, while the other 50% were considered non-urgent [2]. In the ED setting, appropriate high-quality care for abdominal pain can result in quantifiable long-term differences in health outcomes, including survival [3]. However, the distinction between types of abdominal pain–“functional” and “organic”–in children is clinically challenging.

Children with functional abdominal pain are not found to have any organic abnormalities, often despite extensive medical workup, including blood work, imaging studies and endoscopic evaluations totaling over $6000 per patient [4]. In contrast, children with organic abdominal pain have an identifiable metabolic, anatomic, infectious, or inflammatory cause [5], whose variable health outcomes are routinely dependent on timely intervention and management [3,6]

The clinical difficulty is particularly evident in the ED setting, where appendicitis is the top surgical emergency and often leads to an indicated intervention.[3, 7–8] Distinguishing appendicitis [6] from functional causes of pain such as constipation highlights the clinical importance to ensure timely, often life-saving, medical or surgical management. If delayed, perforated appendicitis [6] increases the risk of peritonitis by nearly five-fold [9] and carries the highest mortality among intra-abdominal conditions seen in the ED setting.

Evidence suggests that disparities of care involving inappropriate triage exist in ED settings, with EDs serving as the safety net for medically underserved populations [10–11]. Black adults are more likely to be triaged to a lower level of care and acuity [12–13], have increased wait times for an ICU bed [14] or evaluation for stroke [14], and lower odds of receiving opiates for headache and back pain complaints [15] or triaging to the coronary care unit [16]. In parallel, Black children have lower odds of undergoing laparoscopic appendectomy [17], being prescribed opiate or non-opiate pain medications [18], and receiving testing for chest pain [19].

Additionally, the preliminary evidence of disparities supports the notion that patients lacking insurance or access to a primary care physician are more likely to present to the ED with more non-urgent cases.[20–23]. In separate studies however, families with low incomes and socioeconomic status (SES) have a higher prevalence of abdominal pain among their children [24–25] with increased pain intensity, based on a self-reported scale [25]. In the context of appendicitis, Black and Hispanic children, as well as children on Medicaid, are more likely to have a perforated appendix as compared to White children [26].

However, no literature exists for children in the ED setting presenting with abdominal pain that describes differences in health outcomes based on race and socioeconomic status (SES).

We aim to achieve the following objectives in this study: 1) to identify disparities in health care outcomes among children presenting to the ED with abdominal pain, based on race and SES and 2) to explain the possible mechanism underlying adverse health outcomes as a result of disparities to ED care.

## Methods

### Study Design

**Fig 1:**
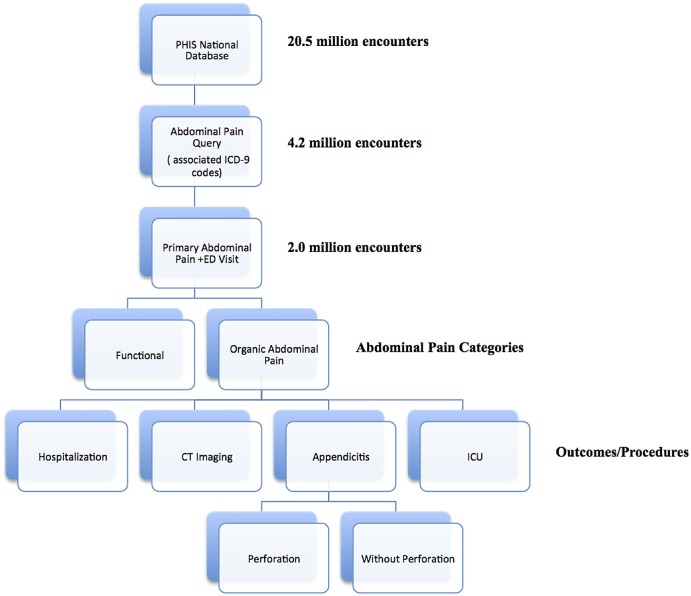
Summary of Study.

We used the Pediatric Health Information System (PHIS), a database of then 43 tertiary children’s hospitals belonging to the Child Health Corporation of America, to query 4.2 million patient encounters (representing 2.7 million pediatric patients (ages 0–30) occurring between January 1, 2004 and December 31, 2011 with discharge diagnoses of abdominal related cases (International Classification of Diseases, Ninth Revision (ICD-9) codes, including 530.x - 579.x, 787.x, 789.x). We further filtered for primary discharge diagnoses of abdominal pain linked to initial ED encounters (2,025,238 encounters, 47.9% of total encounters queried). Fig 1 summarizes the subject identification and filter process within our dataset prior to the analysis phase. We categorized the remaining encounters into functional and organic abdominal pain (functional: 307.7, 564.x, 787.6 789.x; organic: all other included ICD-9 codes such as 276.51, 530.81, 540.9, 558.9, 682.5). Most commonly, these organic abdominal pain diagnoses included urinary tract infection, dehydration, diarrhea, esophageal reflux, cellulitis, appendicitis, and unspecified gastritis and gastroduodenitis, and pyelonephritis. The Stanford University Institutional Review Board exempted this study from full board review since all patient data were de-identified, precluding any risk to any patient-identifiable information.

### Explanatory Variables

Our explanatory variables for all of our logistic regressions and Cox proportional hazard ratios were race/ethnicity, median household income, age, gender, payer type, hospital geographical region, and common chronic childhood diseases. Race/ethnicity categories included White, Black, Hispanic, Asian, and other. Household income category (SES) was based on the median household income generated by the US Census Data from 2010 (GeoLytics Dataset (East Brunswick, NJ) as defined by the patient’s primary zip code, community level statistics that have been shown to represent the individual accurately as a proxy in hospital utilization studies [27]. Our study focused on the highest and lowest median income quartiles with quartile 1 (low income) as ≤$32,214 and quartile 4 (high income) as ≥$52,917. Age was categorized in clinically significant intervals (0–5, 6–12, 13–18, ≥19). Payer types were Public (Medicare, Medicaid, Title V, other governmental sources), Private (Worker’s Compensation, HMO, PPO, TRICARE, other insurance company), and Other (Self-pay and remaining insurance strata). Hospital geographical region categories were Northeast, Midwest, South, Southwest, and West. Based on the literature, binary variables of the following chronic childhood conditions were also captured for the past medical history: history of mental illness, diabetes, cystic fibrosis, obesity, malnutrition, prematurity, asthma, cerebral palsy, and non-specific developmental disability [28].

### Primary Outcome Measures

Our outcome variables for our logistic regressions were ICU admissions, primary diagnoses of appendicitis (ICD-9 540.x-543.x) and perforated appendix (ICD-9 540.0, 540.1), hospital admissions, and CT, MRI, and ultrasound imaging. Our outcome variables for our Cox proportional hazards models were time to surgery and time to discharge from the hospital, beginning with ED presentation.

### Statistical Analyses

We performed all analyses with Stata Statistical Software, version 12 SE (College Station, TX).

#### Chi-squared test

We used the chi-squared-test to detect differences in proportions within patient variables, such as SES levels and race categories. A p-value <0.05 was considered to be statistically significant, and we considered significant variables as explanatory variables in our later analyses.

#### Multivariate logistic regressions

Multivariate logistic regressions were used to determine the effect of explanatory variables (race/ethnicity, median household income, age, gender, payer type, hospital geographical region, and common chronic childhood diseases) on the primary outcome measures (ICU, primary diagnosis of appendicitis, perforated appendicitis, hospital admissions, and surgery). Each outcome variable was tested for potential interaction between the variables race and SES. The data was drawn from encounters from the emergency department with a primary diagnosis of abdominal pain, and all analyses were performed on the patient encounters. When evaluating outcome variables with a complication of appendicitis, appendicitis was used as the denominator. CT, MRI, and ultrasound imaging were performed as a post hoc analysis.

#### Extended Multivariate Cox proportional models

We created two multivariate Cox proportional models to analyze time to surgery and time to discharge home. Time was noted to start from the ED visit and explanatory variables were the same as the logistic regressions as described above. We also tested the proportional hazards assumption via a log-log graph and graphed Schoenfeld residuals for race and income, and we have included an example in the supplementary figures (S1 and S2 Figs).

### Appendicitis as a Proxy for Organic Abdominal Pain

As mentioned in the introduction, appendicitis is a common organic cause for ED evaluations in infants and children. Clinically relevant, appendicitis represents a major diagnostic differential to consider for acute abdominal pain in the ED, requiring urgent or emergent abdominal surgery. Health outcomes in appendicitis are dependent on early detection and appropriate management. Thus, we assumed appendicitis with and without perforation to be a proxy for organic abdominal pain needing acute medical care. We also assumed that perforated appendicitis (i.e., appendicitis associated with generalized peritonitis or peritoneal abscess (ICD-9, 540.0, 540.1)) to be a natural disease sequelae of delayed surgical intervention from delayed patient presentation, delayed care, or previously missed diagnosis. We also assumed that patients with surgeries within the first two days of their encounter who had a primary diagnosis of appendicitis received an appendectomy.

## Results

### Patient Characteristics

Among the 4,227,129 abdominal pain encounters in the composite dataset from 2004 to 2011, 2,926,551 (69.2%) included an ED visit and 2,025,238 (47.9%) included an ED visit with primary diagnosis of abdominal pain, including appendicitis (2.9%). Table 1 summarizes the patients included in the dataset. The median yearly household income was $40,460. Grouped together, minority patients (43.4%, Black and Hispanic) constituted a nearly equal proportion to Whites (45.7%) in our dataset. While all regions of the US are represented, hospitals from the Midwest (31.7%) and South (25.1%), reported the highest proportion of encounters. Age distribution was similar for whites and blacks, but Hispanics had a greater number of patients in the 0–5 and 6–12 age group, rather than a greater percentage of older children.

**Table 1:** Patient Demographics (Patient Encounters of Abdominal Pain).

	Encounters	%	Unique Patients	%
**TOTAL**	4,227,129		2,698,692	
**GENDER**				
Male	2,045,118	48.4	1,311,089	48.6
Female	2,181,830	51.6	1,389,235	51.5
**RACE/ETHNICITY**				
White	1,944,955	47.6	1,232,862	45.7
Black	937,481	22.9	590,922	21.9
Hispanic	859,644	21.0	578,901	21.5
Asian	73,785	1.8	51,387	1.9
Other	272,424	6.7	198,880	7.4
**PAYER**				
Public	2,134,716	51.7	1,350,498	50.0
Private	1,359,269	32.9	940,983	34.9
Self-Pay/Other	633,026	15.3	480,481	17.8
**AGE**				
0–5	2,292,036	54.2	1,519,639	56.3
12-Jun	1,040,997	24.6	729,633	27.0
13–18	770,836	18.2	490,910	18.2
> = 19	123,255	2.9	77,156	2.9
**MEDICAL OUTCOMES**				
Appendicitis	118,144	2.9	110,792	4.1
Death	17,493	0.4	17,487	0.6
ED	2,926,551	69.2	2,074,065	76.9
ED Readmissions^1^	217,743	5.2	158,798	7.7
Hospitalization	1,434,933	34.0	932,240	34.5
Hopsitalization Readmissions^2^	69,342	1.6	58,017	6.2
ICU	209,350	5.0	156,739	5.8
OR Visit	462,135	10.9	369,536	13.7
**PAST MEDICAL HISTORY**				
Asthma	808,061	19.1	361,469	13.4
Obesity	108,397	2.6	41,290	1.5
Diabetes	75,154	1.8	33,277	1.2
Cystic Fibrosis	38,713	0.9	9,217	0.3
Malnutrition	153,486	3.6	34,266	1.3
Cerebral Palsy	152,685	3.6	40,825	1.5
Prematurity	19,212	0.5	5,664	0.2
Mental Illness	226,231	5.4	73,420	2.7
**HOSPITAL REGION**				
Northeast	559,004	13.5	356,403	13.2
Midwest	1,313,080	31.7	781,619	29.0
South	1,039,987	25.1	681,546	25.3
Southwest	506,940	12.3	348,169	12.9
West	720,822	17.4	472,828	17.5

^1^ Calculated as percentage of those in ED

^2^ Calculated as percentage of those in hospital

### Minorities and Low Income Have Lower Hospital Observations and Less Imaging

**Fig 2:**
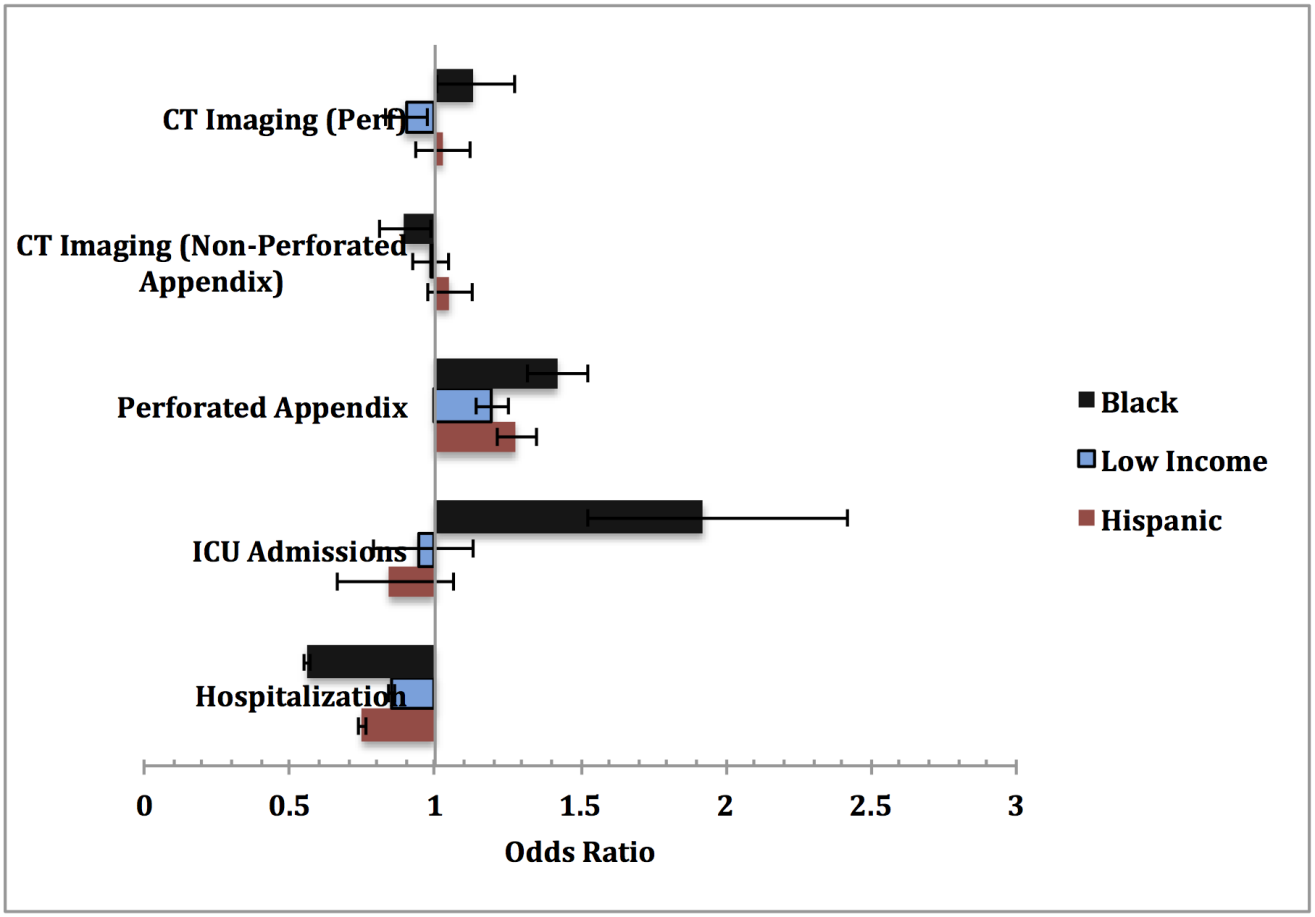
Adjusted Odds Ratios Stratified by SES and Clinical Outcomes.

Hospitalization rates from the ED for a primary diagnosis of abdominal pain (either organic or functional) were lower among Blacks (12.5%) or Hispanics (16.4%) than Whites (21.0%), although among the hospitalized, Blacks, Hispanics, and low income children were diagnosed with organic abdominal pain at approximately equal or higher proportions when compared to Whites and high income children (Tables 2 and 3). We also further stratified by race within income brackets for these various medical outcomes (S1 Table). Minorities (Blacks and Hispanics) and low income children had lower odds of hospital admissions than Whites, even after controlling for all primary diagnoses of organic abdominal pain (Fig 2, Table 4). Additionally, of those presenting to the ED, Blacks and Hispanics had significantly lower odds of radiologic imaging for organic abdominal pain, including computerized tomography (CT), magnetic resonance imaging (MRI), and ultrasound scans (data not shown). Low income children with organic abdominal pain also had lower odds of CT scans as compared to high income children presenting to the ED.

**Table 2:** Patient Diagnoses & Outcomes Stratified by Race from the ED.

	White	%	Black	%	Hispanic	%
**ABDOMINAL PAIN**						
Organic	543,345	46.1	342,136	45.1	355,204	53.1
Functional	259,695	22.0	162,569	21.5	139,149	20.8
**APPENDICITIS**						
Total	45,680	3.9	8,924	1.2	29,836	4.5
With Perforation^1^	13,406	29.3	3,293	36.9	12,333	41.3
Hospitalization	168,494	21.0	63,038	12.5	81,081	16.4
ICU	41,764	3.5	20,097	2.7	13,962	2.1

^1^Percentage calculated as a fraction of patients with appendicitis.

**Table 3:** Patient Outcomes Stratified by SES from the ED.

	**High Income** ^2^	**%**	**Low Income** ^3^	**%**
**ABDOMINAL PAIN**				
Organic	331,196	46.7	383,012	49
Functional	158,294	22.3	160,635	20.6
**APPENDICITIS**				
Total	30,282	4.3	19,757	2.5
With Perforation^1^	9,163	30.3	7,805	39.5
Hospitalization	101,794	20.8	82,468	15.2
ICU	24,356	3.4	18,938	2.4
	**High Income** ^2^	**%**	**Low Income** ^3^	**%**
**ABDOMINAL PAIN**				
Organic	331,196	46.7	383,012	49
Functional	158,294	22.3	160,635	20.6
**APPENDICITIS**				
Total	30,282	4.3	19,757	2.5
With Perforation^1^	9,163	30.3	7,805	39.5
Hospitalization	101,794	20.8	82,468	15.2
ICU	24,356	3.4	18,938	2.4

^1^Percentage calculated as a fraction of patients with appendicitis.

^2^High Income is defined as greater than top quartile income of $52,917.

^3^Low Income is defined as below the bottom quartile income of $32,214.

**Table 4:** SES Influence on Hospital Outcomes.

	Adjusted OR	95% CI
**PERFORATED APPENDIX**		
White ^1^	—	—
High Income (≥$52,917)^2^	—	—
Black	1.42	(1.32–1.53)
Low Income (<$32,214)	1.20	(1.14–1.25)
Hispanic	1.28	(1.21–1.35)
**NON-PERFORATED APPENDIX**		
White^1^	—	—
High Income^2^	—	—
Black	0.29	(0.28–0.30)
Low Income	0.77	(0.75–0.79)
Hispanic	1.12	(1.08–1.16)
**ICU ADMISSIONS**		
White^1^	—	—
High Income^2^	—	—
Black	1.92	(1.53–2.42)
Low Income	0.94	(0.79–1.13)
Hispanic	0.84	(0.67–1.06)
**CT IMAGING (Perforation)**		
White^1^	—	—
High Income^2^	—	—
Black	1.13	(1.00–1.27)
Low Income	0.90	(0.83–0.97)
Hispanic	1.03	(0.93–1.13)
**CT IMAGING (Non-Perforated)**		
White^1^	—	—
High Income^2^	—	—
Black	0.90	(0.81–0.99)
Low Income	0.98	(0.93–1.05)
Hispanic	1.05	(0.98–1.13)
**HOSPITALIZATION**		
White^1^	—	—
High Income^2^	—	—
Black	0.56	(0.55–0.57)
Low Income	0.85	(0.84–0.86)
Hispanic	0.75	(0.74–0.76)

^1^Whites are compared to Blacks and Hispanics.

^2^High Income is compared to Low Income.

### Minorities and Low Income Have Less Surgery, Increased Perforated Appendicitis, and Longer Hospital Stays

In an adjusted Cox proportional hazards model, following admittance through the ED, Blacks (aHR, 1.28, 95% CI 1.27–1.30) and Hispanics (aHR, 1.26, 95% CI 1.24–1.27) had nearly 30% higher likelihood of being discharged earlier for their organic abdominal pain than White patients. (Table 5). However, using appendicitis with perforation as a proxy for serious organic abdominal pain, Blacks (36.9%), Hispanics (41.3%), and low income children (29.3%) had lower rates of non-perforated appendicitis, but higher rates of appendicitis with perforation than Whites and high income children. Similarly, Blacks (aOR, 0.29, 95% CI 0.28–0.30) and low income children (aOR, 0.77, 95% CI 0.75–0.79) had lower odds of a primary diagnosis of non-perforated appendicitis but at least 20% increased odds of perforated appendicitis (aOR, 1.42, 95% CI, 1.32–1.53; aOR, 1.20, 95% CI 1.14–1.25) as compared to Whites and high income children (Table 4).

**Table 5:** Time to Discharge from the ED and Hospital.

	Adjusted HR	95% CI
**ORGANIC ABDOMINAL PAIN**		
White and High Income^1^	—	—
White or High Income^2^	—	—
Black	1.28	(1.27–1.30)
Low Income	0.90	(0.89–0.91)
Black + Low Income	1.11	(1.09–1.12)
Hispanic	1.26	(1.24–1.27)
**TOTAL APPENDICITIS**		
White and High Income^1^	—	—
White or High Income^2^	—	—
Black	0.91	(0.86–0.96)
Low Income	0.89	(0.86–0.92)
Black + Low Income	0.85	(0.79–0.91)
Hispanic	0.99	(0.96–1.03)
**PERFORATED APPENDIX**		
White and High Income^1^	—	—
White or High Income^2^	—	—
Black	0.90	(0.83–0.99)
Low Income	1.04	(0.99–1.10)
Black + Low Income	0.94	(0.84–1.05)
Hispanic	0.84	(0.67–1.06)
**NON-PERFORATED APPENDIX**		
White and High Income^1^	—	—
White or High Income^2^	—	—
Black	1.00	(0.94–1.07)
Low Income	0.85	(0.82–0.89)
Black + Low Income	0.90	(0.83–0.98)
Hispanic	1.05	(1.00–1.11)

^1^White and High Income is compared to Black and Low Income.

^2^White is compared to Black or Hispanic. High Income is compared to Low Income.

Blacks (aHR, 0.74, 95% CI 0.59–0.93), Hispanics (aHR, 0.75, 95% CI 0.75–0.90) and low income children (aHR 0.64, 95% CI 0.55–0.75) were at least 25% less likely of having surgery with their appendicitis at any time point than Whites and high income patients respectively. Low income patients (aHR, 0.61, 95% CI 0.48–0.77) were 39% less likely even with appendicitis with perforation to receive surgery at any time point. Blacks (aOR, 1.92, 95% CI 1.53–2.42) had more complex cases of appendicitis, with nearly two-fold higher odds of an ICU admission. They spent almost 10% longer in the hospital for their appendicitis than Whites (aHR, 0.91, 95% CI 0.86–0.96) (Table 5), even after controlling for a primary diagnosis of perforated appendicitis (aHR, 0.90, 95% CI 0.83–0.99).

### Combined Impact of Race and SES Is Greater than Either One Separately

When we evaluated race and income together, we found that the magnitude was greater than race or income alone (Table 6). Low income Blacks had 63% lower odds of receiving CT imaging for organic abdominal pain (aOR, 0.37, 95% CI 0.36–0.39) and 10% lower odds of CT imaging for non-perforated appendicitis (aOR, 0.86, 95% CI 0.76–0.97), magnitudes lower than either race or income alone. Low income Blacks also had decreased odds of hospitalization (aOR, 0.45, 95% CI 0.44–0.46) and non-perforated appendicitis but 65% increased odds for perforated appendicitis (aOR, 1.65, 95% CI 1.50–1.81), again larger than race or income alone.

**Table 6:** Combined effect of SES and Race on Hospital Outcomes.

	Adjusted OR	95% CI
**PERFORATED APPENDIX**		
White and High Income	—	—
Black and Low Income	1.65	(1.50–1.81)
**NON-PERFORATED APPENDIX**		
White and High Income	—	—
Black and Low Income	0.21	(0.20–0.22)
**ICU ADMISSIONS**		
White and High Income	—	—
Black and Low Income	1.97	(1.47–2.64)
**CT IMAGING (Perforation)**		
White and High Income	—	—
Black and Low Income	0.99	(0.85–1.15)
**CT IMAGING (Non-Perforated)**		
White and High Income	—	—
Black and Low Income	0.86	(0.76–0.97)
**HOSPITALIZATION**		
White and High Income	—	—
Black and Low Income	0.45	(0.44–0.46)

**Fig 3:**
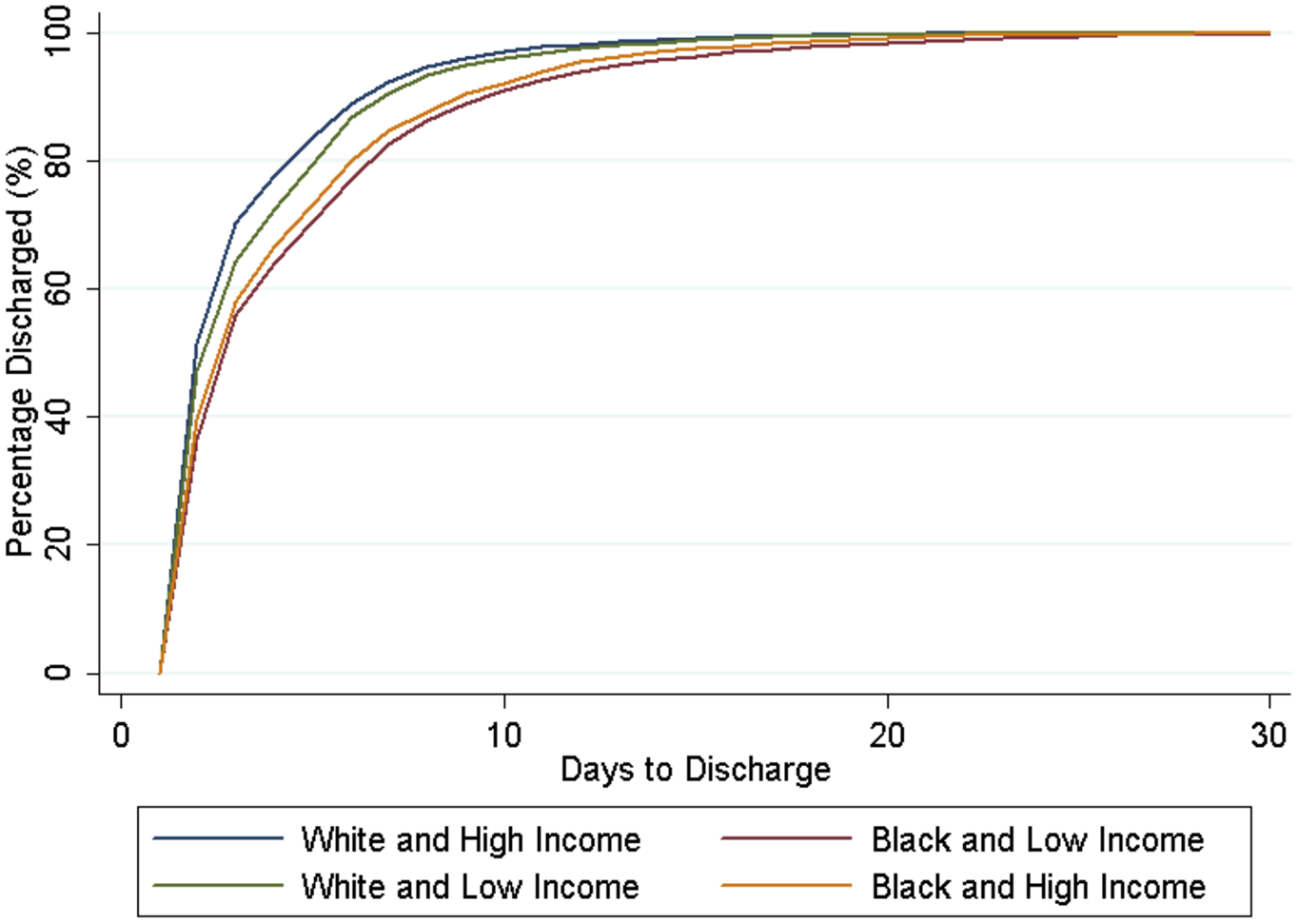
Days Until Discharge Home after Appendicitis.

Low income Blacks had more severe cases of appendicitis, with 15% delayed discharges for total appendicitis (aHR, 0.85, 95% CI 0.79–0.91) (Table 5, Fig 3), including 10% for specifically non-perforated appendicitis (aHR, 0.90, 95% CI 0.83–0.98). They also had 97% increased odds of ICU admission for appendicitis (aOR, 1.97, 95% CI 1.47–2.64) and had lower odds of receiving surgery at any given time point when compared to high income Whites. In a post hoc analysis, we excluded children ages 0–5 because they were less likely to get appendicitis. The magnitude of the results increased and the trend was the same.

### High Income Blacks Have Less Hospitalization and Worse Outcomes than Low Income Whites

**Fig 4:**
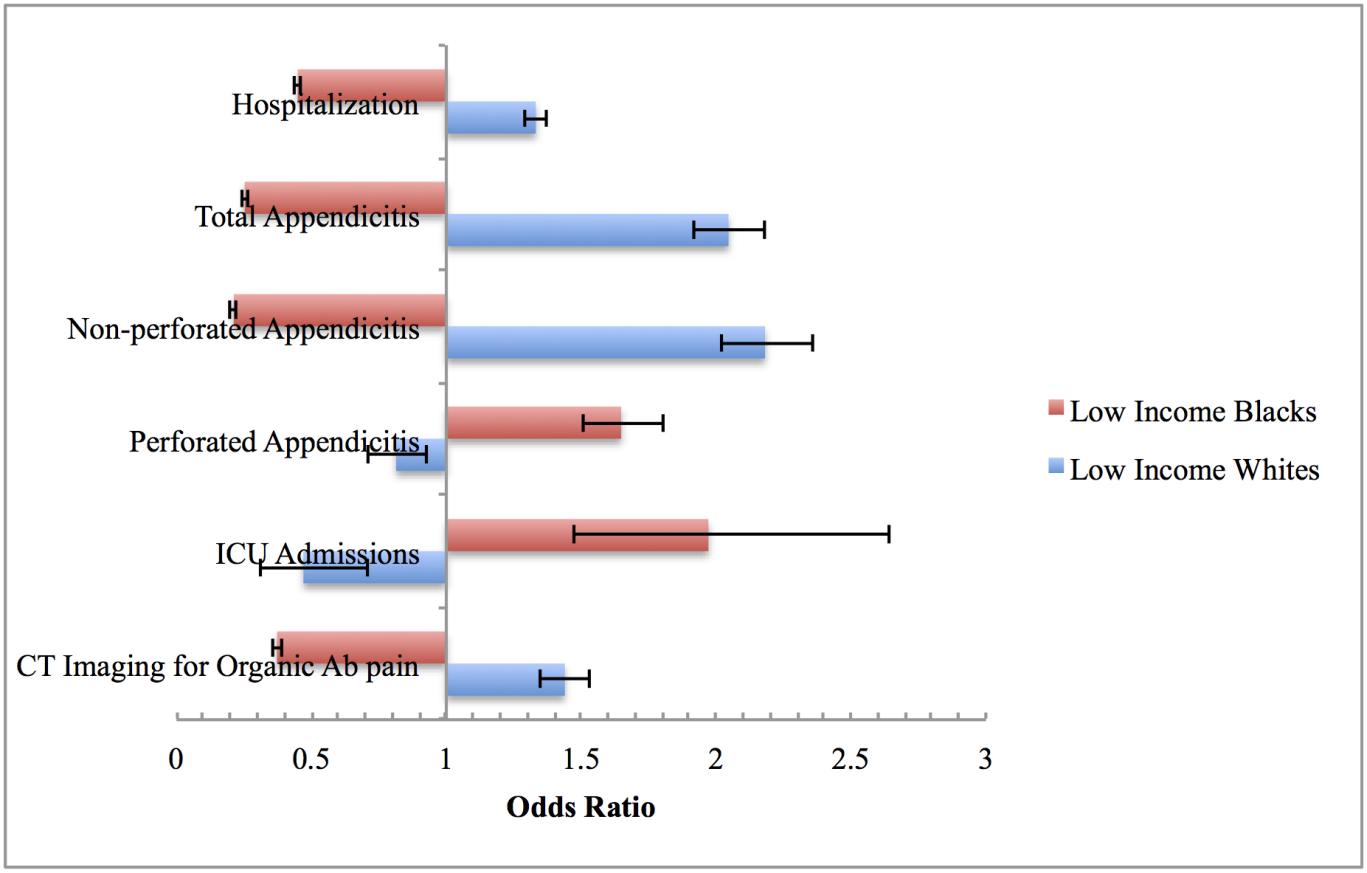
Interaction between Race and Income among Black and Whites. ^1^Low Income Blacks compared with High Income Whites. ^2^Low Income Whites compared with High Income Blacks.

**Fig 5:**
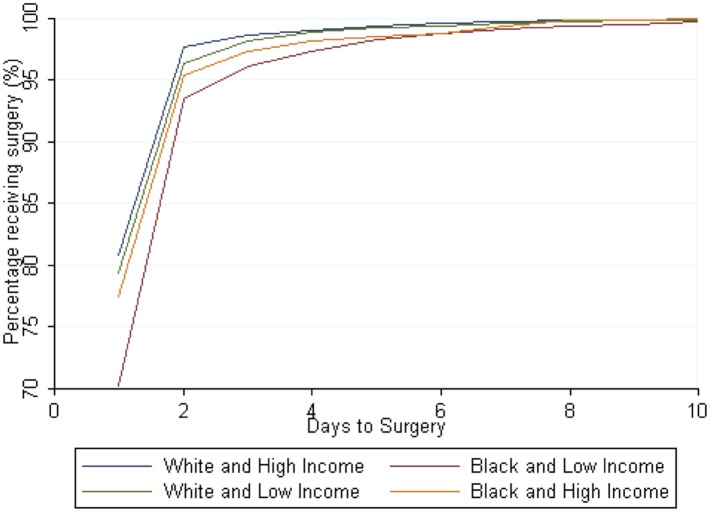
Days to Surgery for General Appendicitis.

Nevertheless, when the opposite scenario is considered—low income Whites compared to high income blacks—low income Whites had increased odds of hospitalization (aOR, 1.33, 95% CI 1.29–1.37) and CT imaging (aOR, 1.44, 95% CI 1.35–1.53) for their organic abdominal pain than high income Blacks (Fig 4). Similarly, low income whites had a higher odds of appendicitis, but lower odds of perforated appendicitis (aOR, 0.81, 95% CI 0.71–0.93) (Fig 4). Low income Whites had higher rates of times to surgery and time to discharge than high income Blacks and low income blacks (Figs 3 and 5).

## Discussion

In a national cohort of 4,229,127 abdominal pain encounters, among them a majority—nearly 3 million—beginning in the ED care setting, we found that race and income differences correlate strongly with health outcomes among US children. First, Blacks, Hispanics, and low income children were less likely to be hospitalized from the ED than Whites—despite either similar or higher proportions of organic abdominal pain in Blacks and Hispanics, respectively. Second, Blacks, Hispanics, and low income children also had lower odds of imaging, including CT scans, for their organic abdominal pain. Third, using appendicitis as a clinical surrogate for organic abdominal pain in the ED, Blacks and Hispanics had higher odds of perforated appendicitis; Blacks had higher odds of ICU admissions and greater length of hospitalization overall. At any time point, Blacks, Hispanics, and low income children were also less likely to get surgery, even with a diagnosis of appendicitis.

Corroborating with our findings, a previous study shows that Blacks have lower odds of developing appendicitis, higher odds of complex appendicitis with perforation, and longer lengths of stay [17]. Additionally, Blacks, Hispanics, and children on Medicaid children are more likely to have perforated appendicitis, regardless of the hospital volume of the site of treatment [26]. However, neither of these studies tracked patients based on their initial presentation and triage in an ED. Our study has implications on the nature of patient care for abdominal pain, especially as the majority of abdominal pain cases in the PHIS dataset began in the ED. Differential care of patients and outcomes based on race and income may also begin here. This finding would have major implications on the ED and its role as a gatekeeper to address marginalized patients.

Based on our investigation, specific policy discussions are important. First, disparity of outcomes exists in the acute ED setting among children presenting with abdominal pain based on race and SES. Although difficult to extrapolate causality, our study supports existing literature on differences in health outcomes in the ED due to race and SES, as summarized by the Institute of Medicine [29]. One investigation concludes that observational stays outnumber 1–day stays from the ED, and patients of either observational or 1-day stays presented with similar medical conditions [30]. Thus, shorter hospitalizations serve similar patient concerns as those simply requiring hospital observation. In our study, Blacks, Hispanics, and low income children had lower odds of hospitalization for organic abdominal pain. Furthermore, we found that Blacks received less radiologic imaging overall for their generalized appendicitis but were equally likely to receive CT imaging for their perforated appendicitis—suggesting that imaging for Blacks was more reserved until urgent medical intervention was necessary, as Blacks had an unexplained 42% higher odds of appendicitis with perforation than Whites. Despite concerns for radiation exposure, safety, and efficacy [31–32], imaging is a highly sensitive test to determine the presence of appendicitis.

Second, differences in care in the ED setting may be attributed to how ED services are generally utilized based on patients’ insurance status and ability to pay for health care at the gatekeeper primary care level. Since ED admissions contain a higher median profit than non-ED admissions, there is an economic incentive to move patients from EDs into a hospital bed [33–34]. Nevertheless, as shown in adolescents, patients use more ED resources when their primary care is lacking or are without health insurance [35], although there is some evidence that seeking non-urgent care in the ED is unrelated to insurance status [36]. In our study, Blacks and Hispanics had significantly fewer health insurance resources, which has been linked to lack of access to a primary care physician for early monitoring of medical conditions as people in poorer health were more than twice as likely to use the ED [37]. Evidence supports that Blacks and Hispanics are historically unable to see ED physicians within triage times comparable to White patients [38], spend longer overall times in the ED [39], and are more likely to be denied authorization for ED visits by managed care organizations [40].

Third, the interpersonal dynamics between the provider and patient are difficult to parse and may play a role in clinical care delivery. In real-time delivery of care, when a child is seen for a chief complaint of abdominal pain, potential language and cultural barriers at the patient-provider level could potentiate suboptimal care among minorities and poor patients, especially in time-sensitive care [29] and over-crowded settings [41] as in the ED [29]. Literature supports language barriers as a major independent risk factor impeding quality health care [42–43]. Behavioral psychology during the delivery of care needs more careful evaluation, as racial differences between a physician and patient (e.g., White physician and Black patient) may be contributing to increased lack of mutual trust to facilitate care [44]. To fill this need for understanding of socioeconomic and race issues for clinicians, there have been programs to raise awareness of potential implicit biases among medical students [45], to understand unconscious biases [46],and to practice habituation with the goal of non-conscious processes to decrease bias [47]. In our study, we felt that focusing on Blacks, Whites, and Hispanics captured a representative population based on the population frequencies. However, Asians also had increased likelihood of appendicitis with perforation, and their results were included in the S2 Table. Race and SES also influence physician perception of patients’ abilities and behavioral tendencies [44]. In our study, it is conceivable that the longer wait times for surgery for Blacks, Hispanics, and low income children could potentiate complicated appendicitis, as non-Hispanic Black children were previously found to have lower odds of non-emergent ED visits than non-Hispanic White children [36].

Our investigation has several limitations. While our PHIS database is composed of 43 tertiary children’s hospitals, there are many of hospitals outside of its realm and it is conceivable that patient demographics are not representative of all pediatric patients. Another limitation is that all codes are bundled based on the ED encounter, not by patient, because our data were structured to allow for breadth of abdominal pain visits. However, we believe the assumption of independence is upheld, as clinically, the diagnosis of appendicitis would likely only be offered once per patient as the management changes with this diagnosis, in addition to major risk and liability to clinician and patient. In our dataset, the vast majority of encounters as appendicitis were unique (< 3% were repeated encounters.) Our analysis involved only primary diagnoses that had presented to the emergency department and the focus was primarily on appendicitis. With bundling based on ED encounter, a procedure completed in the ED is not separated from a procedure in the hospital, if the patient is ultimately hospitalized. In addition, our PHIS database includes a variable of time since ED presentation, and not time since symptoms of abdominal pain. Finally, as discussed previously, beyond an associative relationship between race/SES and health outcomes related to organic abdominal pain, especially appendicitis severity, we are not able to conclusively remark on etiological processes in differential care. The goal of our analysis was to hypothesis test the relationship of race and income to various outcomes of abdominal pain after controlling for potential confounders. As an extension, we wanted to test the predictive properties of our model. We calculated the Hosmer –Lemeshow goodness of fit test statistic for our models, with chi-squared <0.05. Our analysis serves our original purpose to relate race and income to outcomes of abdominal pain, but it does not serve as a predictor model. Furthermore, the Hosmer Lemeshow goodness of fit test statistic has a known dependence on sample size for its power, and therefore our large sample sizes likely greatly contributed to the test statistic.

In conclusion, our investigation is the first and largest of its kind to explore health outcome disparities among children in the ED with abdominal pain. Although our analysis may be describing one microcosm of the more macro-level disparities that may be occurring outside the ED—for example, primary care access for all children—it serves as quantifiable evidence to highlight the differential care and outcomes that exist in the health care system associated with non-disease patient attributes.

## Supporting Information

S1 Fig Log Log Plot of Appendicitis with Perforation Race, Income Combined (Time to Discharge). (TIFF)

S2 Fig Schoenfeld Residual Appendicitis with Perforation Race, Income Combined (Time to Discharge). (TIFF)

S1 Table Patient Outcomes Stratified by SES from the ED. (DOCX)

S2 Table SES Influence on Hospital Outcomes (Aggregate). (DOCX)
