# Supplementary figures and images for: Health Outcomes in US Children with Abdominal Pain at Major Emergency Departments Associated with Race and Socioeconomic Status
Source: PLoS One. 2015 Aug 12;10(8):e0132758. doi: 10.1371/journal.pone.0132758 (PMC4534408; doi:10.1371/journal.pone.0132758)

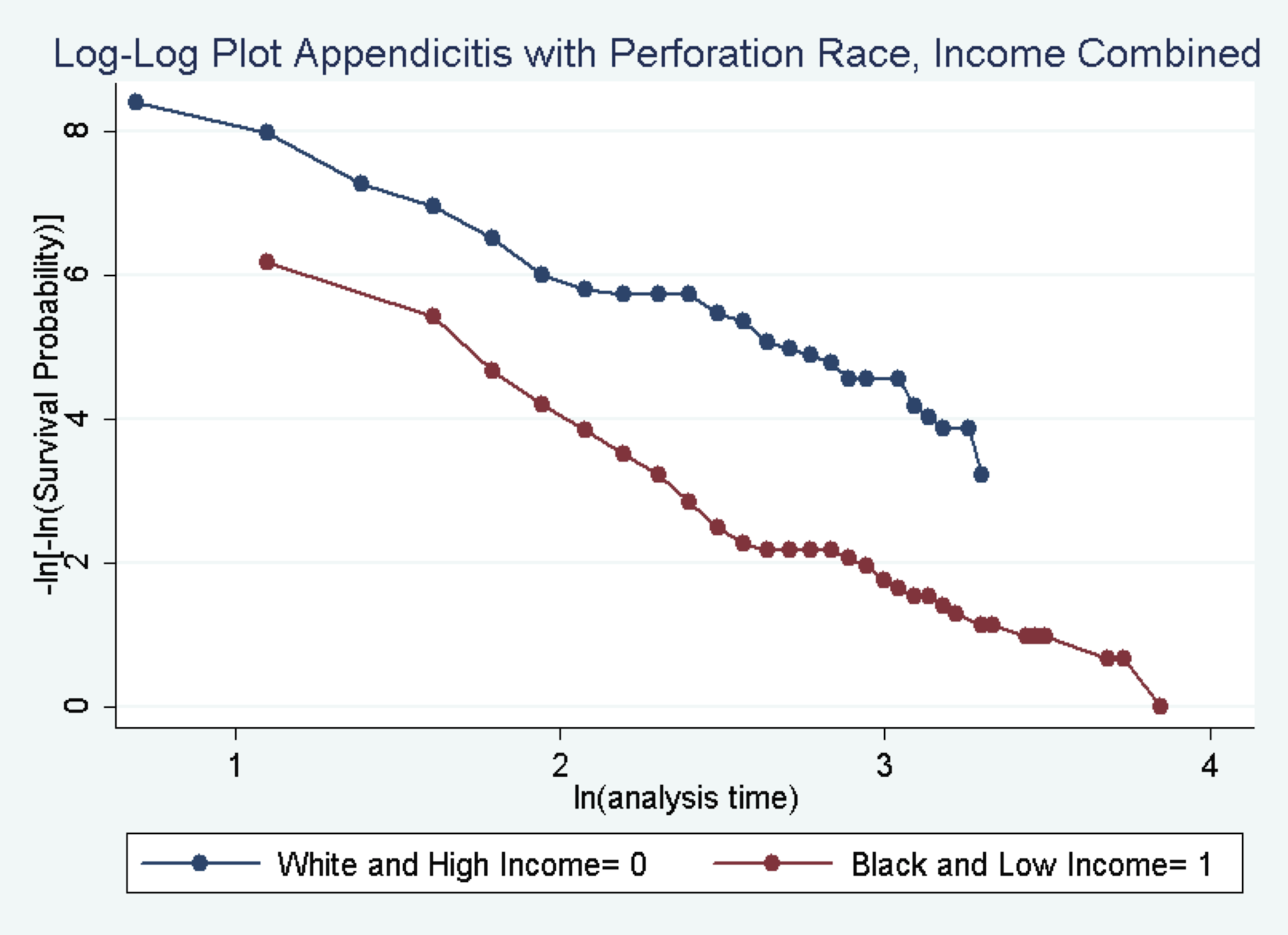

Supplement: S1 Fig — (TIFF) [file pone.0132758.s001.tiff]

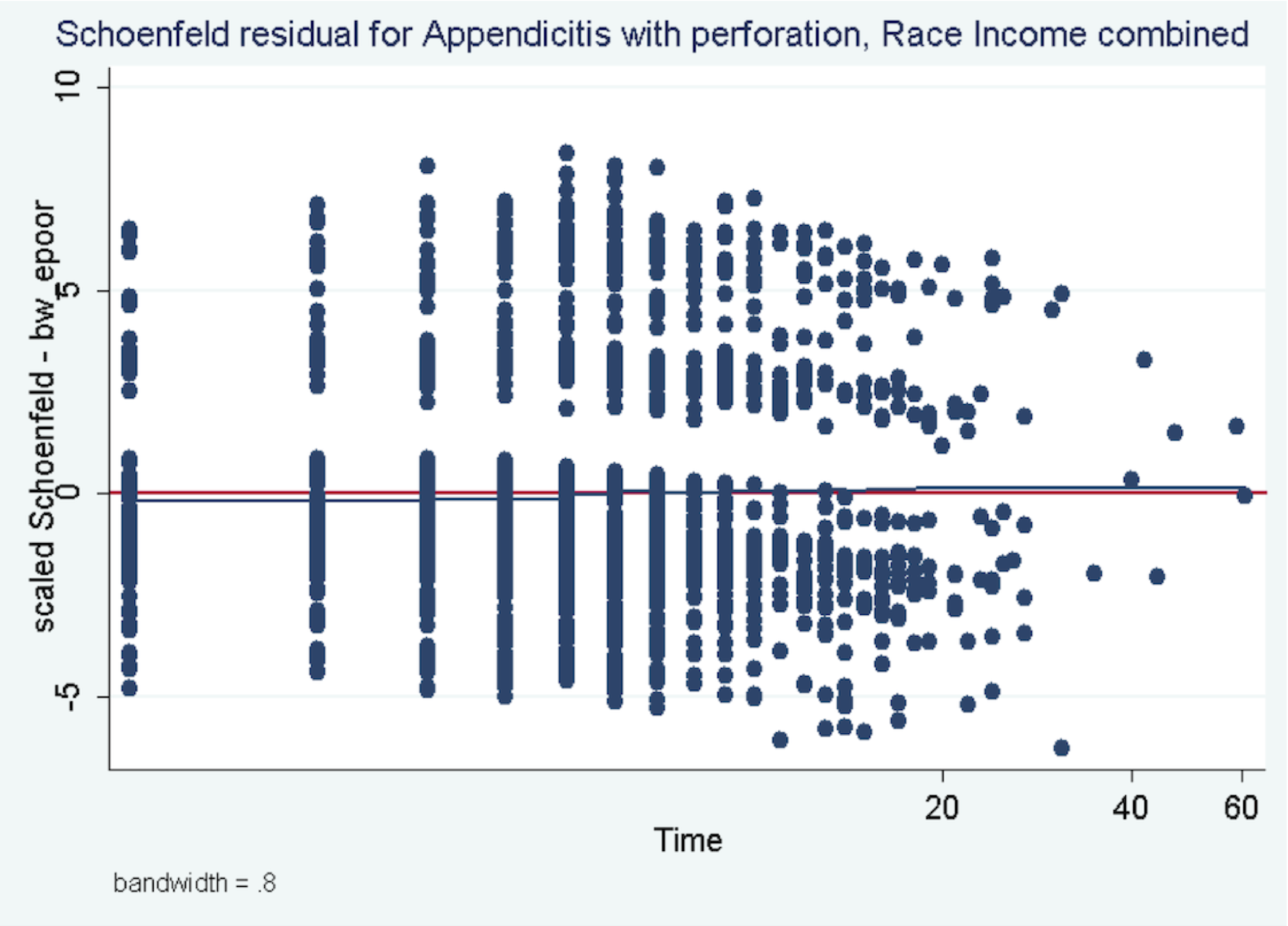

Supplement: S2 Fig — (TIFF) [file pone.0132758.s002.tiff]
