# Supplementary material for: Health Outcomes in US Children with Abdominal Pain at Major Emergency Departments Associated with Race and Socioeconomic Status
Source: PLoS One. 2015 Aug 12;10(8):e0132758. doi: 10.1371/journal.pone.0132758 (PMC4534408; doi:10.1371/journal.pone.0132758)
Supplement: S1 Table — (DOCX) [file pone.0132758.s003.docx]

|  |  | **High Income^1^** | **%** |  | **Low Income^2^** | **%** |
| --- | --- | --- | --- | --- | --- | --- |
| **ABDOMINAL PAIN** |  |  |  |  |  |  |
| **Organic** |  | 331,196 |  |  | 383,012 |  |
|  | White^3^ | 184,921 | 34.0 |  | 82,269 | 15.1 |
|  | Black | 41,135 | 12.0 |  | 157,345 | 46.0 |
|  | Hispanic | 63,078 | 17.8 |  | 103,370 | 29.0 |
| **Functional** |  | 158,294 |  |  | 160,635 |  |
|  | White^3^ | 94,618 | 36.4 |  | 34,628 | 13.3 |
|  | Black | 19,782 | 12.2 |  | 72,054 | 44.3 |
|  | Hispanic | 24,908 | 17.9 |  | 38,179 | 27.4 |
| **APPENDICITIS** |  |  |  |  |  |  |
| **Total** |  | 30,282 |  |  | 19,757 |  |
| **With Perforation^4^** |  | 9,163 | 30.3 |  | 7,805 | 39.5 |
|  | White | 5,311 | 39.6 |  | 1,679 | 12.5 |
|  | Black | 546 | 16.6 |  | 1,303 | 39.6 |
|  | Hispanic | 2,039 | 16.5 |  | 3,730 | 30.2 |
| **Hospitalization** |  | 101,794 |  |  | 82,468 |  |
|  | White | 63,360 | 37.6 |  | 23,342 | 13.9 |
|  | Black | 9,918 | 15.7 |  | 25,496 | 40.5 |
|  | Hispanic | 15,096 | 18.6 |  | 23,750 | 29.3 |
| **ICU** |  | 5,186 |  |  | 3,594 |  |
|  | White | 3,160 | 36.8 |  | 1,178 | 13.7 |
|  | Black | 710 | 20.1 |  | 1,269 | 35.9 |
|  | Hispanic | 574 | 19.4 |  | 725 | 24.4 |

**S1 Table. Patient Outcomes Stratified by SES from the ED.**

| ^1^High Income is defined as greater than top quartile income of $52,917. |
| --- |
| ^2^Low Income is defined as below the bottom quartile income of $32,214. |
| ^3^Percentage of patient encounters meeting both medical outcome and race subcategory, stratified by income. |
| ^4^ Percentage calculated as a fraction of patients with appendicitis. |
