# Supplementary material for: Health Outcomes in US Children with Abdominal Pain at Major Emergency Departments Associated with Race and Socioeconomic Status
Source: PLoS One. 2015 Aug 12;10(8):e0132758. doi: 10.1371/journal.pone.0132758 (PMC4534408; doi:10.1371/journal.pone.0132758)
Supplement: S2 Table — (DOCX) [file pone.0132758.s004.docx]

|  | **Adjusted OR** | **95% CI** |
| --- | --- | --- |
| **PERFORATED APPENDIX** |  |  |
| White and/or High Income^1^ | — | — |
| Black | 1.42 | (1.32 - 1.53) |
| Low Income | 1.20 | (1.14 - 1.25) |
| Black and Low Income | 1.65 | (1.50 - 1.81) |
| Hispanic | 1.28 | (1.21 - 1.35) |
| Asian | 1.34 | (1.17 - 1.54) |
| **APPENDICITIS** |  |  |
| White and/or High Income^1^ | — | — |
| Black | 0.32 | (0.31 - 0.33) |
| Low Income | 0.85 | (0.83-0.87) |
| Black and Low Income | 0.25 | (0.24 - 0.26) |
| Hispanic | 1.24 | (1.21 - 1.28) |
| Asian | 1.07 | (1.00 - 1.15) |
| **NON-PERFORATED APPENDIX** |  |  |
| White and/or High Income^1^ | — | — |
| Black | 0.29 | (0.28 – 0.30) |
| Low Income | 0.77 | (0.75 – 0.79) |
| Black and Low Income | 0.21 | (0.20 – 0.22) |
| Hispanic | 1.12 | (1.08 – 1.16) |
| Asian | 0.96 | (0.88 – 1.04) |
| **ICU ADMISSIONS** |  |  |
| White and/or High Income^1^ | — | — |
| Black | 1.92 | (1.53 - 2.42) |
| Low Income | 0.94 | (0.79 - 1.13) |
| Black and Low Income | 1.97 | (1.47 - 2.64) |
| Hispanic | 0.84 | (0.67 - 1.06) |
| Asian | 1.79 | (1.15 - 2.79) |
| **CT IMAGING (Perforation)** |  |  |
| White and/or High Income^1^ | — | — |
| Black | 1.13 | (1.00 - 1.27) |
| Low Income | 0.90 | (0.83 - 0.97) |
| Black and Low Income | 0.99 | (0.85 - 1.15) |
| Hispanic | 1.03 | (0.93 - 1.13) |
| Asian | 1.20 | (0.97 - 1.49) |
| **CT IMAGING (Non-Perforated)** |  |  |
| White and/or High Income^1^ | — | — |
| Black | 0.90 | (0.81 - 0.99) |
| Low Income | 0.98 | (0.93 - 1.05) |
| Black + Low Income | 0.86 | (0.76 - 0.97) |
| Hispanic | 1.05 | (0.98 - 1.13) |
| Asian | 0.82 | (0.68 - 1.00) |
| **HOSPITALIZATION** |  |  |
| White and/or High Income^1^ | — | — |
| Black | 0.56 | (0.55 - 0.57) |
| Low Income | 0.85 | (0.84 - 0.86) |
| Black + Low Income | 0.45 | (0.44 - 0.46) |
| Hispanic | 0.75 | (0.74 - 0.76) |
| Asian | 1.15 | (1.10 - 1.20) |

**S2 Table. SES Influence on Hospital Outcomes (Aggregate).**

| ^1^White and High Income is compared to Black and Low Income. |
| --- |
| White is compared to Black or Hispanic. High Income is compared to Low Income. |
